# Protective Effect of N-Acetylcysteine (NAC) on oxLDL-Induced Endothelial Dysfunction

**DOI:** 10.4014/jmb.2504.04039

**Published:** 2025-08-05

**Authors:** Chathuri K. Marasinghe, Indyaswan Tegar Suryaningtyas, Won-Kyo Jung, Jae-Young Je

**Affiliations:** 1 Department of Food and Life Science, Pukyong National University, Busan 48513, Republic of Korea; 2 Research Center for Marine-Integrated Bionics Technology, Pukyong National University, Busan 48513, Republic of Korea; 3 Research Center for Food Technology and Processing, National Research and Innovation Agency, Yogyakarta 55861, Indonesia; 4 Major of Biomedical Engineering, Division of Smart Healthcare, Pukyong National University, Busan 48513, Republic of Korea; 5 Major of Human Bioconvergence, Division of Smart Healthcare, Pukyong National University, Busan 48513, Republic of Korea

**Keywords:** Endothelial dysfunction, *N*-acetylcysteine, oxidative stress, oxLDL, cardiovascular diseases

## Abstract

*N*-acetylcysteine (NAC), a well-known antioxidant and glutathione precursor, has been extensively studied for its free radical-scavenging properties, anti-inflammatory effects, and ability to enhance cellular redox balance. NAC has also been shown to mitigate oxidative damage in various disease models, yet its role in endothelial dysfunction remains underexplored. In this study, we evaluated the ability of NAC to counteract oxLDL-induced endothelial dysfunction in human umbilical vein endothelial cells (HUVECs). NAC treatment significantly reduced ROS levels, lipid peroxidation, and apoptotic markers while restoring mitochondrial membrane potential (MMP) and NO bioavailability. Additionally, NAC regulated the expression of eNOS, LOX-1, ICAM-1, and VCAM-1, demonstrating its role in reducing endothelial inflammation and improving vascular homeostasis. Furthermore, NAC prevented excessive cholesterol accumulation, suggesting its potential to regulate lipid metabolism in endothelial cells. These findings highlight the therapeutic potential of NAC in protecting against oxLDL-induced endothelial dysfunction and preventing vascular complications associated with cardiovascular diseases.

## Introduction

Cardiovascular diseases (CVDs) continue to be the primary cause of death globally, with endothelial dysfunction recognized as a critical trigger for vascular pathologies [1, 2]. Maintaining vascular homeostasis depends heavily on the endothelium, which regulates key functions such as blood flow, vascular tone, coagulation, and immune surveillance through its single-cell layer lining the blood vessels [3, 4]. A healthy endothelium produces nitric oxide (NO), a critical vasodilator that maintains proper vessel function, while also suppressing inflammation and oxidative stress [5]. However, when the endothelium is exposed to chronic stressors, such as oxidized low-density lipoprotein (oxLDL), reactive oxygen species (ROS), and inflammatory cytokines, its function becomes impaired, leading to vascular dysfunction and structural damage [6, 7].

A dysfunctional endothelium is marked by diminished NO levels, elevated oxidative stress, persistent inflammation, and increased vascular permeability, collectively contributing to the advancement of atherosclerosis, hypertension, and thrombotic events [1, 7]. A major contributor to this process is oxLDL, which disrupts endothelial homeostasis by promoting ROS generation, inducing mitochondrial dysfunction, and triggering inflammatory signaling pathways [6, 8]. This oxidative burden not only damages endothelial cells but also stimulates the upregulation of adhesion molecules, such as ICAM-1 and VCAM-1, facilitating leukocyte adhesion and chronic vascular inflammation [9, 10]. Additionally, lipid metabolism dysregulation, particularly cholesterol ester and free cholesterol accumulation in endothelial cells, promotes foam cell formation, an early hallmark of atherosclerotic plaque development [7, 11]. Over time, these pathological changes contribute to vascular stiffening, arterial narrowing, and ultimately, cardiovascular complications such as stroke and myocardial infarction.

Given the central role of endothelial dysfunction in CVD progression, targeting endothelial protection is a promising strategy for disease prevention and management. Antioxidant and anti-inflammatory agents have attracted interest for their ability to mitigate oxidative stress, preserve endothelial integrity, and restore NO production [12-14]. Meanwhile, *N*-acetylcysteine (NAC) has garnered interest in cardiovascular research due to its potent antioxidant and anti-inflammatory properties [15, 16]. Initially applied as a mucolytic in the treatment of acetaminophen toxicity, NAC also serves as a key precursor to glutathione (GSH), a vital intracellular antioxidant [17-20]. Its cardioprotective effects have been demonstrated in multiple disease models, where it attenuates oxidative stress, inhibits inflammatory cytokines, and modulates vascular function [21-24]. Given NAC’s antioxidant, anti-inflammatory, and lipid-modulating capabilities, it remains a promising candidate for preventing endothelial dysfunction and mitigating cardiovascular risk. However, more extensive clinical studies are required to confirm its role as a therapeutic agent in cardiovascular health. Our objective in this study was to assess how NAC counteracts oxLDL-induced endothelial dysfunction by evaluating its impact on oxidative stress, cell death pathways, mitochondrial integrity, NO synthesis, inflammation, and lipid balance. By elucidating these mechanisms, this research provides valuable insights into NAC’s potential role in preventing endothelial dysfunction and mitigating CVD risk.

## Material and Methods

NAC, oxidized low-density lipoprotein (oxLDL), CuSO_4_, MTT reagent, and JC-1 dye were obtained from Sigma-Aldrich (USA). Endothelial cells derived from human umbilical veins (HUVECs, PCS-100-010TM) were sourced from ATCC and grown using the EGM-2 medium system (Lonza, USA), specifically formulated to support endothelial cell proliferation and function. Primary antibodies for Bax, Bcl-2, eNOS, LOX-1, cytochrome c, cleaved caspase-3, ICAM-1, and VCAM-1 were purchased from Santa Cruz Biotechnology (USA). ELISA kits used to assess CAT, GPx, and SOD activities were obtained from Cayman Chemical (USA) and Biomax (Republic of Korea). Apoptotic cell analysis was conducted via flow cytometry using the Annexin V-FITC/PI Detection Kit (BD Biosciences, USA) on a BD FACSCalibur system.

### Oxidized LDL Preparation and Measurement

LDL (1 mg/ml) was oxidized with CuSO_4_ (10 μM) for 4 h at 37°C. Oxidation was confirmed by measuring conjugated diene formation at 234 nm and assessing thiobarbituric acid-reactive substances (TBARS) as an indicator of lipid peroxidation.

### Cell Culture and Treatment

HUVECs were grown in endothelial growth medium containing 10% fetal bovine serum (FBS) and 1% penicillin-streptomycin, and incubated at 37°C in a humidified atmosphere with 5% CO_2_. For experimental procedures, HUVECs were incubated with NAC at 10 or 200 μM for 1 h prior to treatment with oxLDL (100 μg/ml) for an additional 24 h. Control cells were left untreated.

### Cell Viability Assay

To evaluate the cytoprotective effect of NAC against oxLDL-induced endothelial injury, HUVECs were pretreated with three concentrations of NAC (10, 100, and 200 μM) for 24 h prior to oxLDL exposure. Cell viability was assessed using the MTT assay, in which MTT reagent (0.5 mg/ml) was added and incubated for 4 h at 37°C. The resulting formazan crystals were dissolved in DMSO, and absorbance was measured at 570 nm using a microplate reader. Cell viability was calculated relative to the untreated control group. All treatments were performed in triplicate, and data represent the mean ± SD from at least three independent experiments. To determine the appropriate concentrations of NAC for subsequent mechanistic studies, 10 and 200 μM were selected as representative low and high effective doses based on this viability screening.

### Assessment of Intracellular ROS

Intracellular ROS levels were measured using a fluorescence-based assay. Cells were loaded with DCFH-DA and incubated at 37°C for 30 min. The resulting fluorescence signal was recorded using a microplate reader set to 488 nm excitation and 525 nm emission wavelengths. The results were normalized to the control group.

### Evaluation of Lipid Peroxidation Activity

Lipid peroxidation was quantified by measuring malondialdehyde (MDA) levels using an MDA assay kit. Following treatment, cell lysates were prepared, and the reaction mixture was incubated at 95°C for 60 min. Absorbance was recorded at 532 nm, and MDA levels were calculated using a standard curve.

### Antioxidant Enzyme Activity Assay

Enzymatic activities of catalase (CAT), glutathione peroxidase (GPx), and superoxide dismutase (SOD) were assessed using commercial ELISA kits. After harvesting the cell lysates, assays were performed following the protocols, and absorbance was recorded with a microplate reader.

### Apoptosis Detection by Flow Cytometry

Cell apoptosis was analyzed through Annexin V-FITC and propidium iodide (PI) double staining, followed by flow cytometric assessment. After collection, cells were rinsed with phosphate-buffered saline (PBS) and incubated with the staining solution for 15 min in the dark. Apoptotic cells were quantified using flow cytometry, and data were analyzed using flow cytometry software.

### Western Blot Analysis

Cell lysates were prepared using RIPA buffer containing protease and phosphatase inhibitors. The mitochondria and cytosol fractions were extracted using a Mitochondria Isolation Kit (Novus Biologicals, Centennial, CO., USA). Protein samples were resolved by SDS-PAGE and transferred onto PVDF membranes, which were then blocked using 5% non-fat milk. Primary antibodies were applied to the membranes and incubated overnight at 4°C, after which HRP-conjugated secondary antibodies were added for detection. Protein bands were visualized using enhanced chemiluminescence (Life Technologies, Republic of Korea), visualized using the Davinch-Chemi Imager (CAS400SM, Core Bio, Republic of Korea), and band intensities were quantified with ImageJ.

### Mitochondrial Membrane Potential (MMP) Assay

MMP was assessed using JC-1 staining (Cayman Chemical Co.). Cells were stained with JC-1 dye for 30 min at 37°C, followed by PBS washing. Fluorescence was then detected using a microplate reader with excitation/emission settings of 488/590 nm for red and 488/525 nm for green signals. The red/green fluorescence ratio was used to determine mitochondrial integrity.

### NO Quantification

NO production was assessed in culture supernatants using the Griess reagent method. After collection, samples were mixed with Griess reagent and incubated for 15 min at room temperature. Absorbance was read at 540 nm, and NO levels were calculated based on a standard calibration curve.

### Cholesterol Measurement

Levels of total, free, and esterified cholesterol were analyzed through a colorimetric detection method provided by BioVision Inc., (USA). Cells were lysed, and cholesterol fractions were measured according to the manufacturer’s protocol. Absorbance was recorded at 570 nm, and cholesterol levels were normalized to total protein concentration.

### Data Analysis

All data are presented as mean ± SD from a minimum of three independent experiments. Statistical comparisons were performed using one-way ANOVA followed by Tukey’s post hoc test, conducted with Sigma Plot 12.0 (Systat Software Inc., USA). Differences were considered statistically significant at *p* < 0.05.

## Results

### NAC Protects HUVECs from oxLDL-Induced Cytotoxicity and Oxidative Stress

**Fig. 1:**
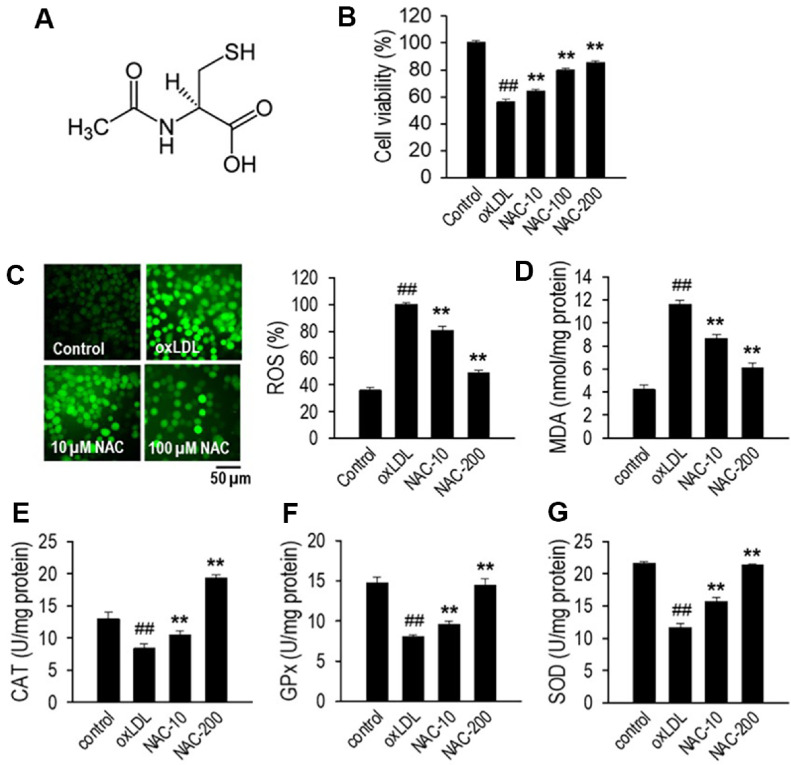
NAC modulates cell viability and oxidative stress in HUVECs exposed to oxLDL. (**A**) Chemical structure of NAC; (**B**) Cell viability assessment: HUVECs were exposed to NAC (10, 100, or 200 μM) for 24 h, or pretreated with NAC for 1 h prior to 24 h oxLDL (100 μg/ml) exposure. Viability was measured using the MTT assay. (**C**) Representative fluorescence images showing ROS levels and corresponding quantification, normalized with protein concentration. (**D**) Quantification of malondialdehyde (MDA) as a marker of lipid peroxidation, assessed using an ELISA kit. (**E-G**) Evaluation of catalase (CAT), glutathione peroxidase (GPx), and superoxide dismutase (SOD) activity in HUVECs. Data are presented as the mean ± SD (*n* = 3). **p < 0.01 compared to the oxLDL-treated group; ^##^*p* < 0.01 compared to the non-treated control group.

To assess how NAC mitigates oxidative stress triggered by oxLDL in HUVECs, we assessed its impact on cell viability, ROS generation, lipid peroxidation (MDA levels), and antioxidant enzyme activity. The chemical structure of NAC is presented in Figure 1A. As shown in Fig. 1B, exposure to oxLDL significantly reduced HUVEC viability by approximately 40% compared to the untreated control (*p* < 0.01). However, NAC treatment at all tested concentrations (10, 100, and 200 μM) significantly restored cell viability (*p* < 0.01), indicating its protective effect against oxLDL-induced cytotoxicity. The selected concentrations were based on previous studies that demonstrated NAC’s cytoprotective and antioxidant effects across a wide range, typically between 1–1,000 μM, depending on cell type and oxidative stress severity [25-27]. In particular, concentrations of 10–200 μM have been frequently used to assess redox modulation and mitochondrial protection in endothelial and vascular models, making them relevant for our aim of evaluating endothelial protection. In addition to reducing cell viability, oxLDL exposure markedly increased ROS generation, as evident from the intense green fluorescence signal in the oxLDL-treated group (Fig. 1C). NAC treatment at 10 and 200 μM significantly reduced ROS fluorescence intensity in a dose-dependent manner (*p* < 0.01), lowering it by up to 50% compared to the oxLDL-only group. To further confirm NAC’s ability to counteract oxidative stress, MDA levels were assessed as a marker of lipid peroxidation. As shown in Fig. 1D, oxLDL exposure led to a significant increase in MDA accumulation (*p* < 0.01), indicating severe oxidative damage. However, NAC treatment effectively suppressed MDA levels, reinforcing its protective role against lipid peroxidation. To determine if NAC boosted the cell’s intrinsic antioxidant capacity, the activities of CAT, GPx, and SOD were analyzed. As shown in Figure 1E-1G, NAC significantly increased the activity of all three enzymes at all tested concentrations (*p* < 0.01), further confirming its role in alleviating oxidative stress.

### NAC Attenuates oxLDL-Induced Apoptosis in HUVECs

**Fig. 2:**
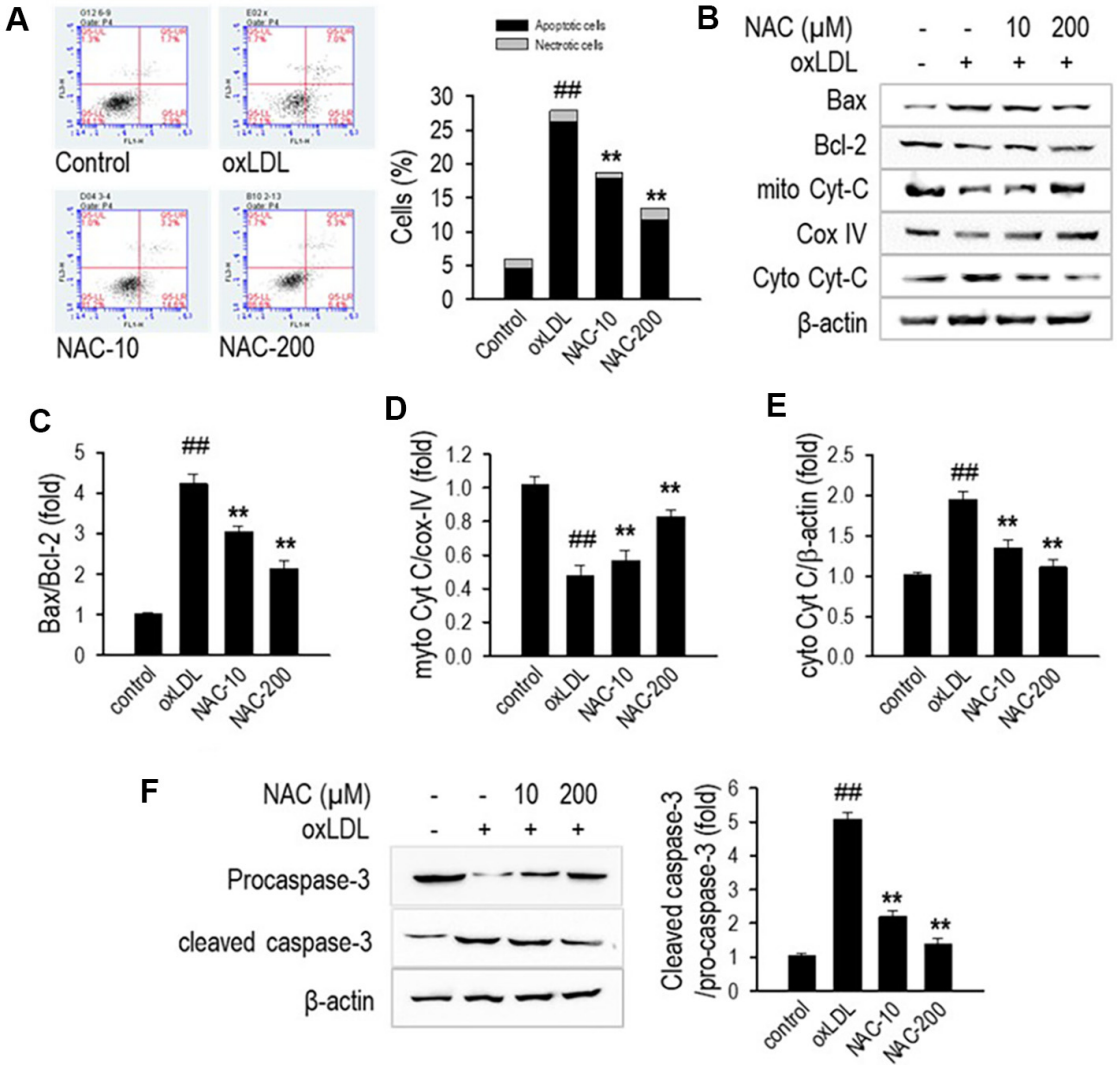
NAC protects HUVECs from oxLDL-induced apoptosis. (**A**) Apoptosis analysis by flow cytometry in HUVECs pretreated with NAC (10 or 200 μM) for 1 h, followed by 24-h exposure to oxLDL. Representative dot plots and quantification of apoptotic cells using Annexin V/PI staining. (**B**) Western blot (WB) analysis of apoptosis-related proteins. (**C**) Quantification of Bax/Bcl-2 ratio. (**D**) Quantification of mitochondrial Cyt-C/Cox-IV. (**E**) Quantification of cytosolic Cyt-C/β-actin. (**F**) Western blot analysis and densitometric quantification of the cleaved caspase-3 to pro-caspase-3 ratio. Proteins were isolated using RIPA buffer containing protease inhibitors, with β-actin serving as the loading control for normalization. Data are presented as the mean ± SD (*n* = 3). **p < 0.01 compared to the oxLDL-treated group; ^##^*p* < 0.01 compared to the non-treated control group.

Exposure to oxLDL triggers marked apoptosis in HUVECs, impairing endothelial function and promoting the development of atherosclerosis. To assess NAC’s protective effects against oxLDL-induced apoptosis, we examined apoptotic cell population, mitochondrial cytochrome c release, and apoptotic protein expression. Flow cytometry analysis with Annexin V/PI staining showed that oxLDL treatment significantly increased the apoptotic cell population to 25%, as indicated by the shift in Annexin V-positive staining (Fig. 2A). However, NAC treatment significantly reduced apoptosis (*p* < 0.01), lowering it to 11.1 ± 0.16% at 200 μM, and 17.8 ± 0.625% at 10 μM, demonstrating its protective effect against oxLDL-induced endothelial cell death.

To further investigate the molecular mechanisms underlying NAC’s anti-apoptotic effects, we assessed key apoptotic markers using western blot analysis (Fig. 2B). The Bax/Bcl-2 ratio, a crucial determinant of apoptosis, was significantly elevated in oxLDL-treated cells, indicating enhanced pro-apoptotic signaling. NAC treatment effectively restored the balance by reducing the Bax/Bcl-2 ratio (Fig. 2C), suggesting an inhibition of the mitochondrial apoptotic pathway. Since Cyt-C release from mitochondria is a hallmark of intrinsic apoptosis, we quantified mitochondrial Cyt-C/Cox-IV and cytosolic Cyt-C/β-actin levels. OxLDL exposure significantly reduced mitochondrial Cyt-C levels (Fig. 2D) while increasing cytosolic Cyt-C accumulation (Fig. 2E), confirming mitochondrial dysfunction and activation of downstream apoptotic signaling. NAC inhibited the release of Cyt-C from mitochondria, thereby maintaining mitochondrial integrity and halting the initiation of apoptosis.

To confirm whether NAC inhibits the execution phase of apoptosis, we assessed caspase-3 activation. Western blot analysis showed that oxLDL significantly increased cleaved caspase-3 expression, indicating apoptotic progression (Fig. 2F). NAC treatment at 10 and 200 μM markedly reduced cleaved caspase-3 expression, as reflected in the cleaved caspase-3/pro-caspase-3 ratio, suggesting that NAC suppresses caspase-3 activation and reinforces its protective role in preventing endothelial apoptosis.

### NAC Preserves Mitochondrial Membrane Potential (MMP) in oxLDL-Treated HUVECs

**Fig. 3:**
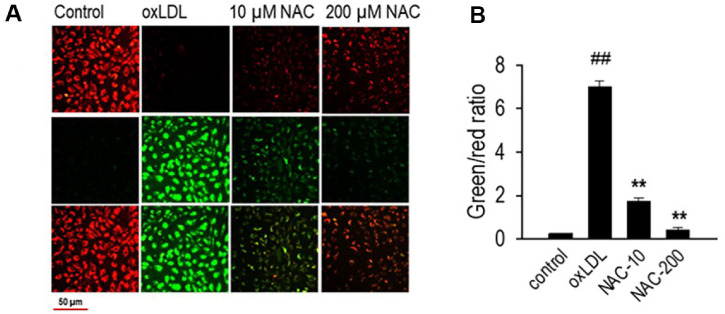
NAC maintains mitochondrial membrane potential integrity. (**A**) JC-1 staining analysis of MMP in HUVECs treated with NAC (10 and 200 μM) for 1 h, followed by oxLDL exposure for 24 h. Red fluorescence reflects functional mitochondria with preserved membrane potential, whereas green fluorescence signals mitochondrial depolarization. (**B**) Quantification of the red/green fluorescence ratio, reflecting MMP integrity. Following the treatments, HUVECs were stained with JC-1 dye following the manufacturer’s protocol, and fluorescence was measured using a fluorescence microscope. Values are expressed as mean ± SD (*n* = 3). **p < 0.01 compared to the oxLDL-treated group; ^##^*p* < 0.01 compared to the non-treated control group.

MMP serves as a critical marker of mitochondrial health, with its loss indicating dysfunction and the onset of apoptosis. To evaluate whether NAC protects mitochondrial integrity, we measured MMP changes using JC-1 staining. As shown in Fig. 3A, oxLDL exposure caused a notable shift from red to green fluorescence, indicating significant mitochondrial depolarization. In contrast, NAC treatment at 10 and 200 μM effectively preserved MMP, as demonstrated by a stronger red fluorescence signal compared to the oxLDL-only group. Quantification of the red/green fluorescence ratio further confirmed this protective effect (Fig. 3B). OxLDL significantly reduced MMP integrity, while NAC treatment restored mitochondrial polarization showing a significant trend with increasing doses (*p* < 0.01).

### NAC Enhances NO Production and Regulates eNOS and LOX-1 Expression

**Fig. 4:**
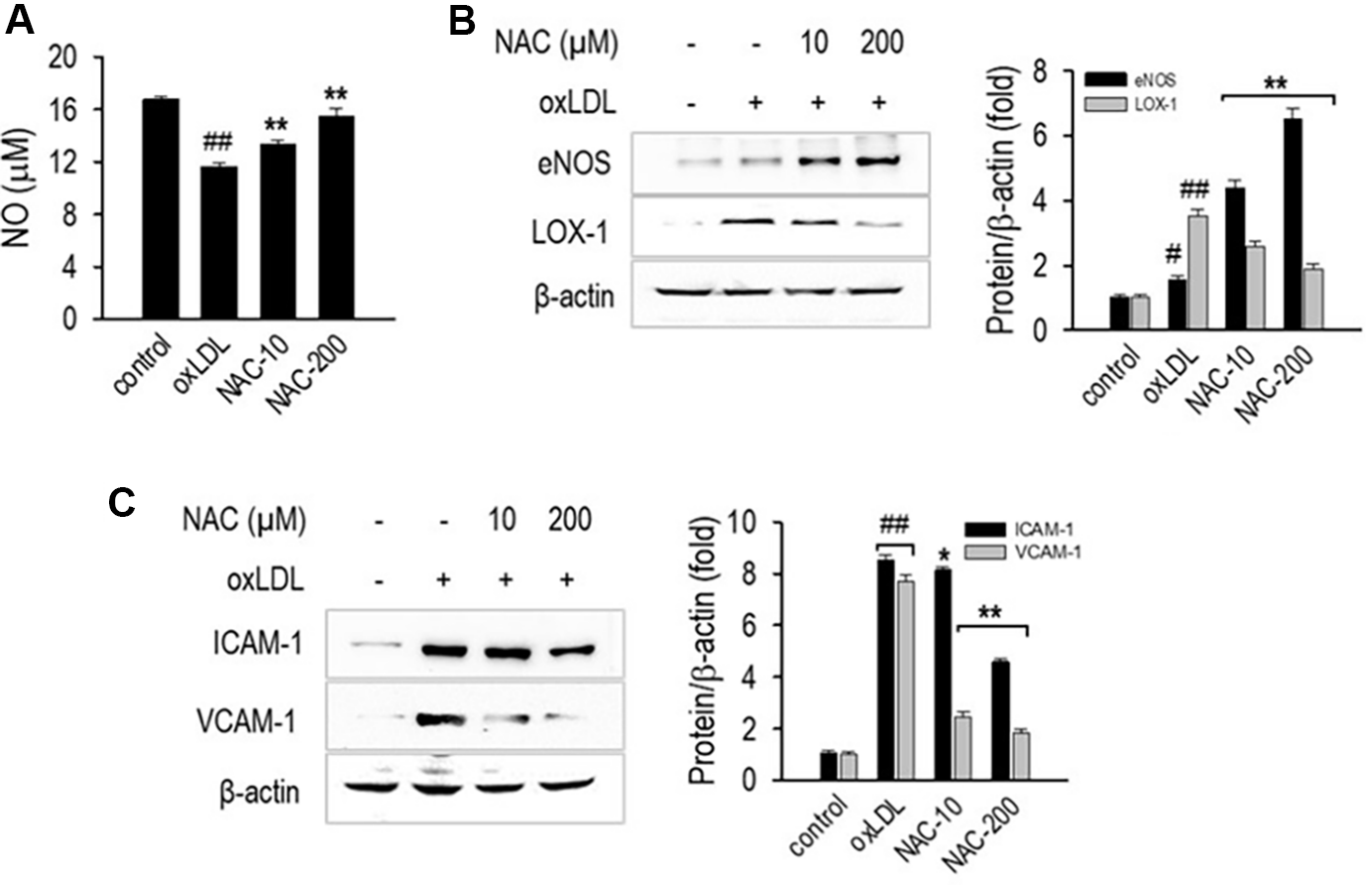
NAC restores NO production and reduces endothelial inflammation. (**A**) NO levels measured using the Griess reagent assay. (**B-C**) Western blot (WB) images of eNOS, LOX-1, ICAM-1, and VCAM-1, with corresponding quantification of fold changes. HUVECs were treated with NAC (10 and 200 μM) for 1 h, followed by oxLDL treatment for 24 h. NO levels in the culture medium were quantified using the Griess reagent assay, following the previously outlined procedure. Western blot analysis was performed to assess the expression levels of eNOS, LOX-1, ICAM-1, and VCAM-1, using β-actin as the internal loading control. Data are presented as mean ± SD (*n* = 3). *p < 0.05, **p < 0.01 versus the oxLDL-treated group; ^##^*p* < 0.05, ^##^*p* < 0.01 versus the untreated control group.

A hallmark of endothelial dysfunction is diminished NO synthesis alongside elevated adhesion molecule expression, both of which contribute to vascular inflammation and disrupted blood flow control. To evaluate whether NAC protects against oxLDL-induced endothelial dysfunction, we assessed NO levels, eNOS and LOX-1 expression, and pro-inflammatory adhesion proteins, including ICAM-1 and VCAM-1. As shown in Fig. 4A, oxLDL treatment significantly reduced NO levels (*p* < 0.01), indicating impaired endothelial function. However, NAC treatment at 10 and 200 μM restored NO production in a dose-dependent manner (*p*<0.01), suggesting its role in preserving endothelial function. Western blot analysis further revealed that oxLDL downregulated eNOS expression while upregulating LOX-1 (Fig. 4B-4C), a receptor that mediates oxLDL-induced oxidative stress. NAC treatment significantly increased eNOS expression while reducing LOX-1 levels, confirming its role in enhancing NO bioavailability and mitigating oxLDL-induced damage.

The upregulation of adhesion molecules such as ICAM-1 and VCAM-1 is a key feature of endothelial inflammation, facilitating leukocyte attachment and contributing to vascular damage. As shown in Figure 4B-4C, oxLDL exposure significantly increased ICAM-1 and VCAM-1 expression, whereas NAC treatment significantly reduced their levels (*p* < 0.01), indicating its ability to suppress oxLDL-induced endothelial inflammation. These findings suggest that NAC protects endothelial cells by restoring NO production, enhancing eNOS expression, reducing LOX-1 expression, and suppressing inflammation via ICAM-1 and VCAM-1 downregulation, thereby preventing oxLDL-induced endothelial dysfunction.

### NAC Modulates Lipid Profile in oxLDL-Treated HUVECs

**Fig. 5:**
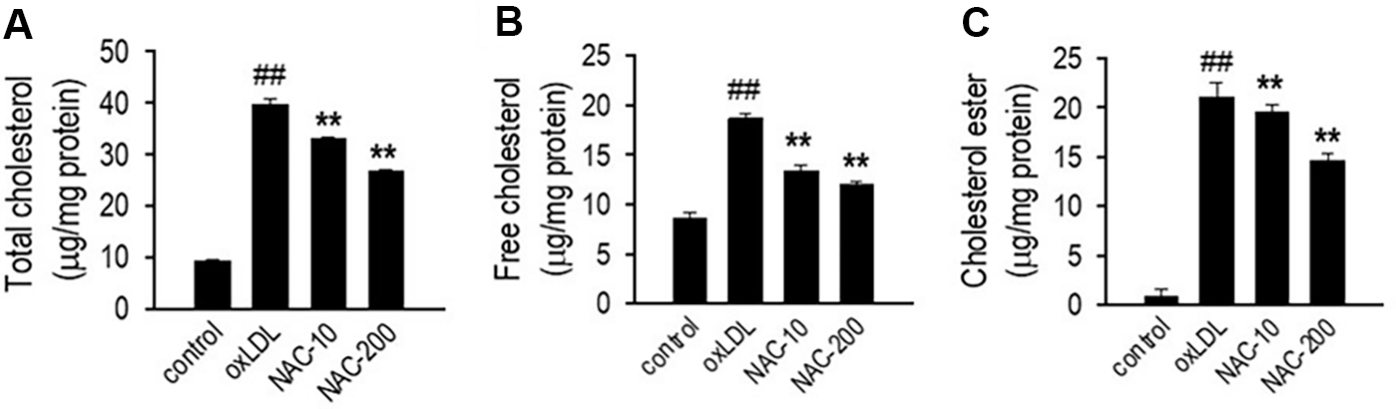
NAC improves lipid profile in oxLDL-treated HUVECs. (**A**) Total cholesterol, (**B**) Free cholesterol, and (**C**) Cholesterol ester levels in HUVECs treated with NAC (10–200 μM) for 1 h, followed by oxLDL exposure for 24 h. HUVECs were plated in 12-well culture plates and incubated for 24 h prior to NAC treatment. Total and free cholesterol levels were quantified using the Total Cholesterol and Cholesteryl Ester Colorimetric Assay Kit II (BioVision Inc., Mountain View, CA) according to the manufacturer’s protocol. Data are expressed as mean ± SD (*n* = 3). **p < 0.01 versus the oxLDL-treated group; ^##^*p* < 0.01 versus the untreated control.

Lipid homeostasis is essential for maintaining endothelial integrity, and its disruption is a key contributor to atherosclerosis progression. OxLDL alters lipid metabolism by increasing cholesterol uptake, impairing efflux, and promoting excessive esterification, which leads to lipid accumulation in endothelial cells. This accumulation contributes to foam cell formation, vascular inflammation, and endothelial dysfunction. To assess the potential of NAC in preventing oxLDL-induced lipid imbalance, levels of total cholesterol (TC), free cholesterol (FC), and cholesterol ester (CE) were measured in HUVECs. As shown in Figure 5A, oxLDL exposure significantly increased TC levels compared to the control group (*p* < 0.01), indicating excessive lipid retention. However, NAC treatment at 10 and 200 μM markedly reduced TC levels in a dose-responsive manner (*p* < 0.01), suggesting its ability to regulate cholesterol balance. A similar trend was observed for FC levels (Fig. 5B), where oxLDL treatment led to a marked increase, while NAC effectively reduced FC accumulation, restoring levels closer to the control. Additionally, oxLDL exposure significantly elevated CE levels (Fig. 5C), contributing to cholesterol storage and foam cell formation, a key step in atherosclerosis development. NAC treatment significantly reduced CE levels (*p* < 0.01), further demonstrating its role in preventing excessive cholesterol esterification.

## Discussion

An imbalance between endothelium-derived vasodilators and vasoconstrictors underlies endothelial dysfunction, a defining feature of vascular disease and a key contributor to the development of atherosclerosis, hypertension, and related cardiovascular conditions [1, 6, 7, 28]. Dysfunctional endothelial cells exhibit reduced NO bioavailability, increased oxidative stress, chronic inflammation, and impaired lipid metabolism, all of which contribute to CVD progression [2]. OxLDL is a major driver of endothelial dysfunction, initiating oxidative stress, stimulating inflammation, causing apoptosis, and disturbing lipid regulation—processes that collectively contribute to vascular injury and atherogenic plaque development [6, 29, 30]. The protective effects of NAC against oxLDL-induced endothelial dysfunction have been investigated in HUVECs, focusing on its ability to counteract oxidative stress, prevent apoptosis, preserve mitochondrial function, regulate NO production, reduce inflammation, and restore lipid balance. As primary endothelial cells, HUVECs closely mimic vascular endothelial behavior, making them an ideal model for investigating oxLDL-induced endothelial dysfunction and potential therapeutic interventions [31, 32]. Previous studies have demonstrated that HUVECs exhibit significant oxidative stress, inflammation, and apoptosis upon exposure to oxLDL, mirroring the endothelial dysfunction observed in atherosclerosis [33-35]. HUVECs provide an effective platform for evaluating NAC’s protective effects against oxLDL-mediated oxidative stress, apoptosis, and inflammation.

OxLDL promotes endothelial dysfunction by interacting with LOX-1, a scavenger receptor that facilitates oxLDL internalization and initiates pro-inflammatory signaling cascades [36-38]. This interaction activates NF-κB, which in turn upregulates inflammatory mediators, including TNF-α, IL-6, and IL-1β, along with adhesion proteins like ICAM-1 and VCAM-1, leading to vascular inflammation and increased endothelial permeability [9, 38] . NAC exerts its protective effect by downregulating LOX-1 expression, thereby reducing oxLDL uptake and inhibiting NF-κB-mediated inflammation. One of the key signaling pathways activated by LOX-1 is NF-κB, a transcription factor that regulates a broad spectrum of inflammatory genes, including cytokines and adhesion molecules. While our study demonstrated that NAC treatment significantly reduced ICAM-1 and VCAM-1 expression—two well-established NF-κB target genes—direct evidence of NF-κB inhibition, such as decreased nuclear translocation of p65 or reduced IκBα phosphorylation, was not assessed [10, 38, 39]. This represents a limitation of our current study. Future investigations should evaluate NAC’s effect on the canonical NF-κB pathway to confirm whether the suppression of endothelial inflammation occurs through direct modulation of NF-κB activity.

Beyond direct ROS scavenging, NAC modulates redox-sensitive signaling pathways such as nuclear factor erythroid 2–related factor 2 (Nrf2) and NF-κB [40, 41]. By elevating intracellular GSH, NAC promotes the release of Nrf2 from Kelch-like ECH-associated protein 1 (Keap1), enabling its nuclear translocation and activation of antioxidant genes like heme oxygenase-1 (HO-1), NAD(P)H:quinone oxidoreductase 1 (NQO1), and glutamate-cysteine ligase catalytic subunit (GCLC), which collectively enhance cellular defense against oxidative stress and restore redox balance [42, 43]. In parallel, NAC suppresses NF-κB activation by inhibiting IκB kinase (IKK)-mediated IκBα degradation, reducing pro-inflammatory cytokine and adhesion molecule expression [44].

OxLDL stimulates excessive ROS production through mitochondrial dysfunction and NADPH oxidase activation, leading to lipid peroxidation, mitochondrial damage, and apoptotic signaling [8, 45] . Mitochondrial damage is a crucial consequence of oxLDL exposure, as it results in MMP loss, Cyt-C release, and caspase activation, triggering endothelial apoptosis [46, 47]. Previous studies have shown that NAC acts as a potent antioxidant by replenishing intracellular GSH and neutralizing ROS, preventing oxLDL-induced lipid peroxidation and mitochondrial dysfunction [48-50]. By preserving mitochondrial integrity, NAC prevents MMP depolarization, thus preventing Cyt-C release and the downstream activation of caspase-3, key events in the apoptotic pathway [51, 52]. Additionally, NAC has been demonstrated to regulate Bax/Bcl-2 balance, promoting cell survival and preventing mitochondrial permeabilization, further reinforcing its role in vascular protection.

Endothelial-derived NO is crucial for vascular homeostasis, as it prevents platelet aggregation, leukocyte adhesion, and smooth muscle proliferation [53, 54]. However, oxLDL exposure reduces NO bioavailability by downregulating eNOS expression and promoting eNOS uncoupling, a process in which eNOS produces superoxide (O_2_^-^) instead of NO, further exacerbating oxidative stress [55, 56]. This shift leads to vasoconstriction, endothelial inflammation, and impaired vascular function [57]. NAC has been shown to enhance eNOS expression and activity, leading to increased NO production and improved endothelial function. Additionally, NAC suppresses LOX-1 expression, reducing oxLDL uptake and limiting eNOS dysfunction, thereby preserving NO bioavailability [36, 37]. Mechanistically, NAC preserves eNOS coupling by elevating intracellular GSH, which plays a critical role in maintaining tetrahydrobiopterin (BH_4_) in its reduced, active form [58, 59]. BH_4_ is an essential cofactor that ensures eNOS produces NO rather than superoxide (O_2_^-^). Under oxidative stress, BH_4_ becomes oxidized, leading to eNOS uncoupling and further ROS generation [59]. By boosting GSH levels, NAC protects BH_4_ from oxidative degradation, thereby stabilizing eNOS dimerization and sustaining NO production. This redox-based preservation of eNOS functionality highlights a key pathway through which NAC restores endothelial homeostasis.

Given the pivotal role of oxLDL in endothelial dysfunction and atherosclerosis, efforts have been made to develop therapeutic strategies targeting oxLDL-mediated damage. Approaches such as scavenger receptor inhibition, lipid-lowering agents, and anti-inflammatory therapies have shown promise in reducing oxLDL-driven endothelial injury . NAC, as a redox-modulating agent, fits within this category due to its ability to neutralize oxidative stress, regulate inflammatory pathways, and prevent endothelial apoptosis. Several preclinical studies have demonstrated NAC’s effectiveness in reducing atherosclerosis progression; however, clinical trials assessing NAC’s cardiovascular benefits have yielded mixed results, with some studies reporting improved endothelial function and others showing limited impact on major cardiovascular outcomes [60, 61]. These discrepancies underscore the need for further investigation into NAC’s optimal dosage, duration of use, and molecular mechanisms of action. From a translational standpoint, the concentrations of NAC used in this study (10–200 μM) are within or close to the range achievable *in vivo*. Oral NAC supplementation (600–1,200 mg/day) typically produces plasma concentrations of 16–35 μM, while intravenous administration can reach up to 300–500 μM, supporting the physiological relevance of our experimental conditions [20, 62]. Moreover, NAC’s favorable safety profile and pharmacokinetic properties enhance its clinical applicability in vascular disease management. This study contributes to addressing these gaps by elucidating the molecular mechanisms through which NAC counteracts oxLDL-induced endothelial dysfunction.

## Conclusion

A central contributor to cardiovascular disease, endothelial dysfunction is largely driven by oxLDL-induced oxidative stress, inflammatory signaling, programmed cell death, and disturbances in lipid metabolism. This study demonstrates that *N*-acetylcysteine (NAC) effectively counteracts oxLDL-induced endothelial injury by scavenging ROS, preserving mitochondrial integrity, inhibiting apoptosis, restoring NO bioavailability, reducing inflammatory signaling, and preventing cholesterol accumulation. These findings highlight NAC’s therapeutic potential in protecting endothelial function and mitigating early vascular damage associated with atherosclerosis and CVDs. While NAC exhibits promising endothelial-protective effects, further research is needed to determine its optimal dosing, long-term efficacy, and clinical applicability in CVD prevention and management.
